# Sarcopenic Obesity in Africa: A Call for Diagnostic Methods and Appropriate Interventions

**DOI:** 10.3389/fnut.2021.661170

**Published:** 2021-04-16

**Authors:** Amy E. Mendham, Lillemor Lundin-Olsson, Julia H. Goedecke, Lisa K. Micklesfield, Dirk L. Christensen, Iain J. Gallagher, Kathryn H. Myburgh, Feyisayo A. Odunitan-Wayas, Estelle V. Lambert, Sebastiana Kalula, Angus M. Hunter, Naomi E. Brooks

**Affiliations:** ^1^SAMRC/Wits Developmental Pathways for Health Research Unit, Department of Paediatrics, School of Clinical Medicine, University of the Witwatersrand, Johannesburg, South Africa; ^2^Health through Physical Activity and Lifestyle Research Centre & Division of Exercise Science and Sports Medicine, Department of Human Biology, Faculty of Health Sciences, University of Cape Town, Cape Town, South Africa; ^3^Department of Community Medicine and Rehabilitation, Umeå University, Umeå, Sweden; ^4^Non-communicable Diseases Research Unit, South African Medical Research Council Tygerberg, Cape Town, South Africa; ^5^Department of Public Health, University of Copenhagen, Copenhagen, Denmark; ^6^Health Sciences and Sport, University of Stirling, Stirling, United Kingdom; ^7^Department of Physiological Sciences, University of Stellenbosch, Stellenbosch, South Africa; ^8^Division of Geriatric Medicine, University of Cape Town, Cape Town, South Africa

**Keywords:** sarcopenia, skeletal muscle, quality of life, aging, muscle function, muscle quality, low and middle-income countries

## Abstract

This perspective aims to highlight the lack of current knowledge on sarcopenic obesity in Africa and to call for diagnostic methods and appropriate interventions. Sarcopenic obesity has been defined as obesity that occurs in combination with low muscle mass and function, which is typically evident in older adults. However, there has been no clear consensus on population-specific diagnostic criterion, which includes both gold-standard measures that can be used in a more advanced health care system, and surrogate measures that can be used in low-income settings with limited resources and funding. Importantly, low and middle-income countries (LMICs) across Africa are in an ongoing state of economic and social transition, which has contributed to an increase in the aging population, alongside the added burden of poverty, obesity, and associated co-morbidities. It is anticipated that alongside the increased prevalence of obesity, these countries will further experience an increase in age-related musculoskeletal diseases such as sarcopenia. The developmental origins of health and disease (DOHaD) approach will allow clinicians and researchers to consider developmental trajectories, and the influence of the environment, for targeting high-risk individuals and communities for treatment and/or prevention-based interventions that are implemented throughout all stages of the life course. Once a valid and reliable diagnostic criterion is developed, we can firstly assess the prevalence and burden of sarcopenic obesity in LMICs in Africa, and secondly, develop appropriate and sustainable interventions that target improved dietary and physical activity behaviors throughout the life course.

## Introduction

This perspective aims to highlight the lack of current knowledge on sarcopenic obesity in Africa and to call for diagnostic methods and appropriate interventions. Importantly, due to increasing urbanization as well as the effective treatment of infectious diseases such as HIV, people in Africa are living longer but presenting with disability and ill health, resulting in an increased burden on family, community and the health care system (1). Increased longevity and the changes in lifestyle behaviors that occur with urbanization, are associated with an increased risk of developing obesity and age-related musculoskeletal diseases such as sarcopenia and osteoporosis (2). Sarcopenia alone contributes to poor health outcomes and frailty in older adults. When sarcopenia occurs in conjunction with obesity, termed “sarcopenic obesity,” it can have further health and functional implications that reduce quality of life and increase overall mortality (3–6) to a greater extent than either condition in isolation (7–10). However, there is a lack of evidence concerning both the prevalence and the burden of sarcopenia and sarcopenic obesity in Africa. It is also unclear as to whether the current diagnostic criteria for these conditions are appropriate for use in African populations. There is a large variability in the prevalence of sarcopenic obesity across LMICs, with India, Ghana, Mexico and South Africa reporting a prevalence of 1.3, 5.4, 10.2, and 10.3%, respectively (11). Although we focus on an African perspective it is hypothesized that the variable prevalence rates reported in the literature may have been significantly influenced by a lack of research and population-specific diagnostic criteria across all LMICs.

## Obesity and Sarcopenia as Comorbid Conditions

Few studies have considered sarcopenia and obesity as comorbid conditions in Africa (11). It is well-established that the aging process stimulates mechanisms resulting in the attenuation of muscle quality (strength or power normalized to muscle size) and muscle mass (12). These mechanisms include reductions in muscle fiber size, fiber number, mitochondrial dysfunction, and contractile function, and increases in ectopic fat accumulation and inflammation within the skeletal muscle (12–15). When age-related loss in muscle quality and quantity occur simultaneously with obesity these mechanisms are amplified and the result is sarcopenic obesity (12). There are also complex interactions between lifestyle behaviors, such as dietary intake and physical activity, and the aging process. All of these collectively contribute to reduced muscle quality, muscle quantity and metabolic health [further explored by (16)].

LMICs across Africa are in an ongoing state of economic and social transition, which has contributed to an increase in the aging population, alongside the added burden of poverty, obesity, and associated co-morbidities (17, 18). Increasing urbanization is also accompanied by a reduction in levels of physical activity, increased sedentary behavior and nutritional deficiencies (19–21). These may occur throughout the life course, and act as risk factors for sarcopenic obesity (15, 22, 23). For example, nutritional deficiency alongside obesity has created a double burden of malnutrition in Africa. Wasting and stunting during early growth and development predisposes individuals to obesity during adulthood (24, 25), potentially placing them at greater risk for the development of sarcopenic obesity as an older adult. Accordingly, we suggest that it is important to consider early life influences, as well as current lifestyle behaviors, to better understand and potentially prevent or mitigate sarcopenic obesity (26, 27) in these settings. The DOHaD approach will allow clinicians and researchers to consider developmental and environmental history for targeting high-risk individuals and communities for treatment and/or prevention-based interventions that can be implemented throughout all stages of the life course. However, the implementation of interventions in Africa are often constrained by limited resources. We urge the research and public health communities to advise on innovative strategies that target lifestyle-related opportunities throughout the life course. Most notably, these strategies need to be co-designed with the communities in which these interventions will be implemented to ensure uptake and sustainability. Interventions and public health initiatives can be subsequently designed to simultaneously target nutritional deprivation, physical inactivity, and obesity throughout the life course in high-risk communities, with the overall aim of preventing sarcopenia, sarcopenic obesity, and associated co-morbidities.

## Begin With the End in Mind: Classification of Sarcopenia and Sarcopenic Obesity

In order to focus on sarcopenic obesity within Africa, it is important to ensure that there are appropriate, population-specific diagnostic criteria to determine more accurate estimates of the disease prevalence and burden. Sarcopenic obesity is the presence of obesity when sarcopenia has been diagnosed. Accordingly, there are two processes that need to be optimized and standardized across populations, firstly the diagnostic criteria for sarcopenia and secondly the classification of obesity. Sarcopenia is a musculoskeletal disease that encompasses a range of diagnostic criteria (28–35). The first definition of sarcopenia was published in 1989, with the main criteria being age-related loss of muscle mass (29). The criteria have since been expanded to include a loss of physical function measured as grip strength and gait speed, with the cut-offs adapted for different populations. Different criteria include the European Working Group on Sarcopenia (EWGSOP) (30), EWGSOP2 (31), Asian Working Group for Sarcopenia (AWGS) (32), International Working Group for Sarcopenia (IWGS) (34), and the Foundation for the National Institutes of Health (FNIH) criteria (35). More locally, Kruger et al. have suggested ethnic-specific cut-points for use in classifying low appendicular skeletal muscle mass index in black South African women (36). Each criterion defines sarcopenia using muscle mass and muscle function indices; however, the diagnostic cut-off values and diagnostic process differs. All these factors have led to variability and inconsistency in reporting the prevalence of sarcopenia, especially in many LMICs where clear diagnostic criteria have not been validated across the diverse populations. A recent assessment of sarcopenia in a Gambian cohort reported that the estimated population prevalence varied according to the criteria applied (37). Specifically, when applying the EWGSOP criteria there was a prevalence of 19% in women and 10% in men, compared to 45% in women and 20% in men when applying the FNIH criteria (37). More, recently, the AWGS presented a clear strategy for the diagnosis and treatment of sarcopenia, with different criteria that can be applied in resource-limited community settings and health care or clinical research settings (32). This is an approach that has been validated across high-income countries (HIC) and LMIC Asian communities, but a similar strategy needs to be implemented to determine whether these criteria can be used across Africa.

The FNIH are the only criteria that incorporate body weight into the sarcopenia criteria as they provide an adjustment for BMI (35). Increased load bearing in obese individuals can lead to greater absolute muscle strength and muscle mass, compared to individuals without obesity (38). However, when muscle strength and muscle mass are normalized to fat mass (i.e., lean to fat mass ratio), obese individuals appear to be weaker and have relatively less muscle (39). We hypothesize that the prevalence of sarcopenia will continually be substantially underestimated until population-specific cut-points (both adjusted and unadjusted for adiposity) are developed to accurately identify low muscle mass and muscle function; similar to that provided by the FNIH. This stems from our recently published data (40) which reported that older South African women (*n* = 122; 60–85 years) from a low-income setting have 3.3% prevalence of sarcopenia without obesity and 24.6% with sarcopenic obesity. We used the BMI adjusted FNIH criteria to firstly diagnose sarcopenia, and the World Health Organization defined categories of BMI to identify obesity. Notably, in this same cohort we reported 4.9, 3.3, 1.6, and 0% with sarcopenic obesity when applying EWGSOP, EWGSOP2, AWGS, and IWGS, respectively. Furthermore, the methods used to classify obesity vary between studies and usually include BMI, fat mass, visceral adipose tissue (%), lean to fat mass ratio and/or waist circumference (41). We suggest that a simple and cost-effective measure such as population-specific BMI cut-points for obesity will suffice in low-income communities. An additional measure of population-specific waist circumference cut-points for central obesity may also be considered. Due to the lack of available literature, Europid cut-points for BMI and waist circumference are used in African populations, with more population specific cut-points needed (42–44). Without a consensus on the diagnostic criteria, it is impossible to accurately measure the prevalence and estimated burden of sarcopenic obesity in Africa. We further suggest that group and country-specific cut-points and diagnostic processes for sarcopenic obesity are needed to avoid additional variability in the classification and reporting prevalence of the disease. Importantly, the criteria should also include both gold-standard measures that can be used in more advanced health care systems and surrogate measures that can be used in low-income settings with limited resources and funding, which is an approach currently being led by the AWGS (32).

## Discussion/Conclusion

The increasing aging population in Africa, together with the challenges of food insecurity and poverty-associated nutrient deficiencies are associated with an increased risk of sarcopenia. Adding to this, the increasing prevalence of obesity places these populations at risk of sarcopenic obesity, and associated risk of overall mortality, co-morbidities, and reduced quality of life. A clear population-specific approach to the diagnostic criteria, treatment and prevention for sarcopenia and sarcopenic obesity is needed to ensure the burden on the health care system and quality of life is not compromised further. Once the prevalence and burden of sarcopenic obesity is established in Africa, there will be a need to develop appropriate and sustainable interventions that target improved dietary and physical activity behaviors throughout the life course.

## Data Availability Statement

The original contributions generated for the study are included in the article/supplementary material, further inquiries can be directed to the corresponding author/s.

## Author Contributions

AM, LL-O, and NB conceptualized this perspective piece and drafted the manuscript. All authors contributed to the article and approved the submitted version.

## Conflict of Interest

The authors declare that the research was conducted in the absence of any commercial or financial relationships that could be construed as a potential conflict of interest.
